# Supplementary material for: Factors related with the incidence of acute respiratory infections in toddlers in Sleman, Yogyakarta, Indonesia: Evidence from the Sleman Health and Demographic Surveillance System
Source: PLoS One. 2021 Sep 24;16(9):e0257881. doi: 10.1371/journal.pone.0257881 (PMC8462707; doi:10.1371/journal.pone.0257881)
Supplement: Attachment — Submitted filename: Response to Reviewers.docx [file pone.0257881.s001.docx]

Dr. Fathmawati Fathmawati

Department of Environmental Health,

Politeknik Kesehatan Kemenkes Pontianak,

Indonesia

Email: fathmawati@poltekkes-pontianak.ac.id or fathmawati.ema@gmail.com

Pontianak, 23 June 2021

Dear Dr. Rahman

Academic Editor PLOS ONE

We appreciate you and the reviewers for your precious time in reviewing our paper titled “Factors related with the incidence of acute respiratory infections in toddlers in Sleman, Yogyakarta, Indonesia: Evidence from The Sleman Health and Demographic Surveillance System” and providing valuable comments. It was your valuable and insightful comments that led to possible improvements in the current version. The authors have carefully considered the comments and tried our best to address every one of them. We hope the manuscript after careful revisions meets your high standards. The authors welcome further constructive comments if any.

Below we provide the point-by-point responses. All modifications in the manuscript have been highlighted in yellow.

**RESPONSES TO ACADEMIC EDITOR**

Here are responses to the improvements we had to make:

1. Please ensure that your manuscript meets PLOS ONE's style requirements, including those for file naming. The PLOS ONE style templates can be found at

https://journals.plos.org/plosone/s/file?id=wjVg/PLOSOne_formatting_sample_main_body.pdf and

https://journals.plos.org/plosone/s/file?id=ba62/PLOSOne_formatting_sample_title_authors_affiliations.pdf

**Response**: Thank you for your kind reminder. We have ensured that the manuscript complies with the PLOS ONE template.

2. Please ensure that you include a title page within your main document. We do appreciate that you have a title page document uploaded as a separate file, however, as per our author guidelines (http://journals.plos.org/plosone/s/submission-guidelines#loc-title-page) we do require this to be part of the manuscript file itself and not uploaded separately.

Could you therefore please include the title page into the beginning of your manuscript file itself, listing all authors and affiliations.

**Response**: Thank you for your kind reminder. The title page has become one file with the manuscript and has listed all the authors and their affiliations.

3. We note that you have indicated that data from this study are available upon request. PLOS only allows data to be available upon request if there are legal or ethical restrictions on sharing data publicly. For information on unacceptable data access restrictions, please see http://journals.plos.org/plosone/s/data-availability#loc-unacceptable-data-access-restrictions.

In your revised cover letter, please address the following prompts:

3a) If there are ethical or legal restrictions on sharing a de-identified data set, please explain them in detail (e.g., data contain potentially identifying or sensitive patient information) and who has imposed them (e.g., an ethics committee). Please also provide contact information for a data access committee, ethics committee, or other institutional body to which data requests may be sent.

3b) If there are no restrictions, please upload the minimal anonymized data set necessary to replicate your study findings as either Supporting Information files or to a stable, public repository and provide us with the relevant URLs, DOIs, or accession numbers. Please see http://www.bmj.com/content/340/bmj.c181.long for guidelines on how to de-identify and prepare clinical data for publication. For a list of acceptable repositories, please see http://journals.plos.org/plosone/s/data-availability#loc-recommended-repositories.

We will update your Data Availability statement on your behalf to reflect the information you provide.

**Response**: Thank you for your kind reminder. Regarding the sharing of data publicly, Sleman HDSS has its policy. Here is the answer from HDSS Sleman Management:

Sleman HDSS is fully funded and conducted by Faculty of Medicine, Public Health and Nursing (FKKMK), UGM. Thus, Sleman HDSS data use follows the terms and conditions implemented by FKKMK UGM. During the first 2 years after data collection, data are only accessible for Sleman HDSS’ researchers and academics community in FKKMK UGM. Afterwards, broader academic communities (e.g., from other universities) are welcome to use Sleman HDSS data. The terms and conditions of Sleman HDSS data use require every user to send the data use application form and brief proposal to Sleman HDSS office for review and to apply for ethical clearance from UGM Medical and Health Research Ethics Committee (MHREC). The applicant will receive the dataset after Sleman HDSS’ reviewers and MHREC approve their application. The data set provided by Sleman HDSS does not include any information that can be used to identify the respondents. Furthermore, the data use terms and conditions also prohibit the data user to make any attempts to identify the respondents.

The main purpose of the “restriction” in Sleman HDSS data use is to protect Sleman HDSS’ respondent personal information. In addition, it also prevents the data use for commercial purposes and prevents duplication in publications based on Sleman HDSS data. Further questions regarding the research ethics of Sleman HDSS data use can be directed to the UGM Medical and Health Research Ethics Committee ([mhrec_fmugm@ugm.ac.id](mailto:mhrec_fmugm@ugm.ac.id) ; **0274 588688 extension 17225 , +62811-2666-869**). Detailed information regarding the procedure of data use and application for data use is available in <https://hdss.fk.ugm.ac.id/> . Further questions can be sent to [hdssjogja.fkkmk@ugm.ac.id](mailto:hdssjogja.fkkmk@ugm.ac.id).

4. Thank you for stating the following in the Acknowledgments Section of your manuscript:

This paper uses data from Sleman HDSS (Health and Demographic Surveillance System) wave 1, 2, 3. The Sleman HDSS data collection has been primarily funded by the Faculty of Medicine, Public Health and Nursing, Universitas Gadjah Mada, Yogyakarta, Indonesia.

We note that you have provided funding information that is not currently declared in your Funding Statement. However, funding information should not appear in the Acknowledgments section or other areas of your manuscript. We will only publish funding information present in the Funding Statement section of the online submission form.

Please remove any funding-related text from the manuscript and let us know how you would like to update your Funding Statement. Currently, your Funding Statement reads as follows:

The author(s) received no specific funding for this work.

Please include your amended statements within your cover letter; we will change the online submission form on your behalf.

**Response**: Thank you for your kind reminder. We do not receive specific funding for this work. This research was conducted independently. We have replaced the Acknowledgments Section in our manuscript with the following sentence:

The authors are grateful to Sleman HDSS Management for allowing us to use Sleman HDSS data; and Klinik Bahasa Faculty of Medicine, Public Health and Nursing UGM for proofreading this manuscript.

**RESPONSES TO REVIEWERS**

Reviewer's Responses to Questions

**Comments to the Author**

1. Is the manuscript technically sound, and do the data support the conclusions?

The manuscript must describe a technically sound piece of scientific research with data that supports the conclusions. Experiments must have been conducted rigorously, with appropriate controls, replication, and sample sizes. The conclusions must be drawn appropriately based on the data presented.

Reviewer #1: Partly

Reviewer #2: Yes

2. Has the statistical analysis been performed appropriately and rigorously?

Reviewer #1: No

Reviewer #2: Yes

3. Have the authors made all data underlying the findings in their manuscript fully available?

The [PLOS Data policy](http://www.plosone.org/static/policies.action#sharing) requires authors to make all data underlying the findings described in their manuscript fully available without restriction, with rare exception (please refer to the Data Availability Statement in the manuscript PDF file). The data should be provided as part of the manuscript or its supporting information, or deposited to a public repository. For example, in addition to summary statistics, the data points behind means, medians and variance measures should be available. If there are restrictions on publicly sharing data—e.g. participant privacy or use of data from a third party—those must be specified.

Reviewer #1: Yes

Reviewer #2: Yes

4. Is the manuscript presented in an intelligible fashion and written in standard English?

PLOS ONE does not copyedit accepted manuscripts, so the language in submitted articles must be clear, correct, and unambiguous. Any typographical or grammatical errors should be corrected at revision, so please note any specific errors here.

Reviewer #1: Yes

Reviewer #2: Yes

5. Review Comments to the Author

Please use the space provided to explain your answers to the questions above. You may also include additional comments for the author, including concerns about dual publication, research ethics, or publication ethics. (Please upload your review as an attachment if it exceeds 20,000 characters)

Reviewer #1:

*1. In the introduction, researchers clearly mentioned the problem statement but did not justify why it is important to conduct this study in a HDSS site; they also did not mention how the HDSS-based study findings will contribute in policy making.*

**Response**: Thank you for pointing this out. We agree with this comment. Therefore, we have added a description of the importance of this research and its benefits for policymakers. This change can be seen in lines 77 - 83.

The results of the 2017 Sleman HDSS showed that ARI was ranked first in infectious diseases in Sleman and the highest ARI cases occurred in children under five [5]. If this condition continues, it will cause impaired growth and development in toddlers. This is the first report from Indonesia using the Sleman HDSS data which examined factors associated with the incidence of ARI in under-five children in Yogyakarta, Indonesia. The findings of this study will aid local governments in developing policies for the management of ARI in children under five.

*2. The experiment was not conducted rigorously. The authors did not mention how they calculated the sample size and why they used the purposive sampling methods in a cross-sectional observational study. Purposive sampling is suitable for qualitative research.*

**Response:** Thank you for pointing this out. We used the entire population of children under five in the Sleman HDSS data that matched the inclusion criteria. We did not include children with exclusion criteria in this study. We have changed the description of the research method. This change is in lines 96 - 99.

This study used a population of children under five suffering from ARI with inclusion and exclusion criteria. Overall, there were 549 children under five suffering from ARI. Only 463 children under five met the inclusion criteria.

*3. In the study design, they mentioned that they analyzed the secondary data from 2015 to 2017 but at the line 63, they mentioned that the study population was from HDSS data in 2017. They need to explain this discrepancy clearly. They also did not mention whether this HDSS site is urban or rural based.*

**Response**: Agree. We have accordingly revised the method section by adding a description of the research area and the data obtained. The changes can be seen in lines 96, 105 - 110.

The Sleman HDSS research area covers urban, sub-urban and rural areas.

Demographic data including characteristics of children under five and maternal characteristics were obtained from HDSS 2017 data. Immunization status and use of mosquito coils were obtained from HDSS 2016 data. Meanwhile, data on house characteristics including type of floor, number of people occupying the house, floor area, house walls, and ceilings, as well as sanitation facilities and water sources were obtained from the 2015 HDSS data.

*4. Except the source of drinking water as a protective factor, the study did not find the significant relationship with any other important predictors like mothers’ education level and occupation, occupancy density, physical condition of house, use of mosquito coil. This study finding is also inconsistent with many other study findings which is not explained properly by the authors. This contradiction may be due to use of inappropriate sampling method. Though authors decided to select the appropriate statistical analysis method but it is not appropriate to run the statistical analysis using a purposive sample in a cross-sectional observational study.*

**Response:** Thank you. For some of the variables, we have added descriptions. However, in terms of occupancy density, we have explained that there is no relationship between occupancy density and the incidence of ARI because only a small proportion of respondents' houses do not meet the standards. Same is the case with the type of wall of the house. We have also revised the methods section on sampling. This study did not take a sample but used data from the entire population of children under five who met the criteria.

We added some explanations of the cause of the absence of a relationship between several variables studied with ARI in children under five. The changes can be seen in

Lines 173 – 175 (for toddler’s sex).

Another study showed that the incidence of other ARI such as tonsillitis and sinusitis was higher in women in all age groups due to differences in the anatomy of the respiratory tract between men and women [24].

Line 184 – 187 (for toddler’s age)

There is no relationship between the age of toddlers and the incidence of ARI in this study because the toddlers involved in this study were aged 25-59 months who have an immune system that is starting to mature [33], so there is no age variation that can distinguish the relationship with ARI.

Line 188 – 195 (for low birth weight)

This study did not find a significant relationship between low birth weight and ARI. A longitudinal study in Bangladesh also found no significant association between birth weight and ARI [22]. However, low birth weight correlates with the incidence of ARI [34]. Some researchers have shown that infants with low birth weight have organs that are not fully developed and are very susceptible to infection [35,36]. There was no relationship between birth weight and ARI in this study due to the few cases of low birth weight found. The Sleman Regency Government seeks to reduce the low birth weight rate by minimizing the anemia rate of pregnant women for the health of mothers and babies [37].

Line 199 – 205 (for immunization)

This study did not show a significant relationship between immunization status and ARI. This is in contrast to studies conducted in two other regions in Indonesia which showed a significant relationship between immunization status and ARI [8,9]. The absence of a relationship between immunization status and ARI may be due to the low immunization coverage in this study. The issue of vaccine rejection in Sleman is undeniable [37,38]. Local governments should prioritize increasing public understanding of vaccines so that immunization coverage can be achieved.

We added some explanations about mother’s education that has no relationship to ARI. The changes can be seen in lines 215 – 219.

The education of the people of Sleman Regency is high. Sleman has a Human Development Index (HDI) higher than the national average. One of the components is the average length of schooling for residents aged 25 years and over which reaches 9.32 years. This figure is higher than the national figure of 8.17 years [39].

We added some explanations about mother’s occupation that has no relationship to ARI. The changes can be seen in lines 229 – 235.

Even though the Sleman Regency Government has issued a regulation requiring workplaces to provide lactation rooms for breastfeeding mothers, some companies have not fully complied with it. This is because business owners do not understand why this regulation was issued or have not received socialization regarding this matter [43]. This study did not have data on exclusive breastfeeding that could support these findings. This finding is in line with previous research which found working mothers have a positive correlation with the incidence of ARI [44].

We added some explanations about occupancy density in line 254 – 255.

A study conducted in Padang, Indonesia found that residential areas with high density were almost 22 times more likely to have ARI in children under five [50].

We added some explanations about ceilings in line 267 – 270.

Both groups have the same opportunity for ARI. The ceiling functions to keep dust from entering the house through the gaps between the walls and the roof. Unfortunately, this study did not have data on indoor air quality.

We revised the explanation about mosquito coils in line 278 – 283

This result is different from previous studies that found a relationship between the use of mosquito coils and ARI [56,57]. The difference between this study and previous studies is the proportion of mosquito coil users in the research subjects. In this study, mosquito coils were not popularly used by respondents, in only about 20%. Meanwhile in previous studies, more than 50% of respondents used mosquito coils.

*5. Authors mentioned that data can’t be shared without the permission of the management team but data which are available in the table is sufficient to understand the study findings.*

**Response:** Thank You

*6. In the conclusion, they did not make any specific recommendation to the policy makers.*

**Response**: We agree with this and revised the conclusion. It can be seen in line 338 – 348.

Although immunization is not related to the incidence of ARI among children under five in Sleman Regency, this finding does not indicate that the immunization program for toddlers in Sleman Regency is successful. However, this is solely due to low immunization coverage. The local government needs to educate residents who refuse their toddlers to be immunized by cooperating with the local community and religious leaders. In addition, the government can provide parenting education to mothers and their families by utilizing health cadres since the mother is pregnant so that the children's health is maintained. The government needs to socialize and enforce regulations regarding exclusive breastfeeding for working mothers by imposing sanctions for companies that have not implemented it. The Sleman Regency Health Office should pay more attention to monitoring the quality of the water used by the community, especially the water produced by the LWC and refill drinking water depots.

*7. Nonetheless the manuscript is presented in an understandable way, still it needs to improve further. Such as:
a. authors mainly compared this study finding with African-based studies instead of Asian countries; it may affect the policy makers to make the applicable decision.*

**Response**: We agree with this comment. Therefore, we have replaced and added some research according to your suggestions.

*b. Typo: prevalence at the line 128, it should be incidence*

**Response**: Thank you. We have fixed it.

*c. Line 152, they mentioned “similar results were reported by [8,9,35] and in Line 201, they mentioned “this research is consistent with [17]; this is not the appropriate way to mention it when they will compare their study findings with other study findings.*

**Response**: Thank you. We have fixed it.

Reviewer #2:

*This article needs to give more information about the reason why some of independent variables was not have significantly related to ARI. Please write argumentation for each factor. This contains is very important to elaborate the condition that different with the other study.*

**Response:** Thank you. We have incorporated your suggestion throughout the manuscript.

We look forward to hearing from you in due time regarding our submission and to respond to any further questions and comments you may have.

Thanks for your kindly.

Best Regards,

Dr. Fathmawati Fathmawati
