# Supplementary material for: Factors related with the incidence of acute respiratory infections in toddlers in Sleman, Yogyakarta, Indonesia: Evidence from the Sleman Health and Demographic Surveillance System
Source: PLoS One. 2021 Sep 24;16(9):e0257881. doi: 10.1371/journal.pone.0257881 (PMC8462707; doi:10.1371/journal.pone.0257881)
Supplement: Attachment — Submitted filename: Response to Reviewers.docx [file pone.0257881.s002.docx]

Dr. Fathmawati Fathmawati

Department of Environmental Health,

Politeknik Kesehatan Kemenkes Pontianak,

Indonesia

Email: fathmawati@poltekkes-pontianak.ac.id or fathmawati.ema@gmail.com

Pontianak, 19 August 2021

Dear Dr. Rahman

Academic Editor PLOS ONE

We have a desire to thank you and the reviewers who allowed us the opportunity to revise our paper entitled "Factors associated with the incidence of ARI in Toddlers in Sleman, Yogyakarta, Indonesia: Evidence from The Sleman Health and Demographic Surveillance System" and provide valuable comments. We think our paper will be better than the previous version after revising according to your precious views and comments. We hope that this manuscript afterward's careful revision meets your high standards. The authors welcome further constructive comments if any.

Below we provide the point-by-point responses. All modifications in the manuscript have been highlighted in yellow.

**RESPONSES TO ACADEMIC EDITOR**

Here are responses to the improvements we had to make:

Journal Requirements:

Please review your reference list to ensure that it is complete and correct. If you have cited papers that have been retracted, please include the rationale for doing so in the manuscript text, or remove these references and replace them with relevant current references. Any changes to the reference list should be mentioned in the rebuttal letter that accompanies your revised manuscript. If you need to cite a retracted article, indicate the article’s retracted status in the References list and also include a citation and full reference for the retraction notice.

**Response:** Thank you for your kind reminder. We have reviewed the references and corrected the retracted papers. To ensure our references are complete and correct, we complete all articles published in the journal with DOI while other references with URLs. In addition, we also added the title of the paper in the original version using Indonesian. We present the details of the changes in the bibliography:

| No. | Number in 2^nd^ version | Number in 3^rd^ version | Changes |
| --- | --- | --- | --- |
| 1. | 1 | 1 | Available: https://www.who.int/publications/i/item/infection-prevention-and-control-of-epidemic-and-pandemic-prone-acute-respiratory-infections-in-health-care |
| 2. | 2 | 2 | Available: https://apps.who.int/iris/handle/10665/324835 |
| 3. | 3 | 3 | Available: https://www.litbang.kemkes.go.id/laporan-riset-kesehatan-dasar-riskesdas/ |
| 4. | 8 | 8 | doi:10.33846/hn.v1i3.59 |
| 5. | 9 | 9 | [Cakupan imunisasi dasar dengan kejadian ISPA pada balita usia 1-3 tahun di wilayah Puskesmas Wonosari 1 Kabupaten Gunung Kidul] |
| 6. | 11 | 11 | [Faktor-faktor yang mempengaruhi kejadian infeksi saluran pernapasan akut (ISPA) pada anak usia 12-59 bulan di Puskesmas Tebet Barat, Kecamatan Tebet, Jakarta Selatan] |
| 7. | 14 | 14 | Tarmure S, Alexescu TG, Orasan O, Negrean V, Sitar-Taut AV, Coste SC, et al. Influence of pesticides on respiratory pathology – A literature review. Ann Agric Env Med. 2020;27: 194–200. doi:10.26444/aaem/121899 |
| 8. | 27 | 27 | doi:10.4103/0974-777X.107167 |
| 9. | 37 | 37 | Available: https://dinkes.slemankab.go.id/download |
| 10. | 38 | 38 | doi:10.37638/jsk.25.3.1-13 |
| 11. | 39 | 39 | Available: https://yogyakarta.bps.go.id/pressrelease/2019/05/06/951/indeks-pembangunan-manusia-d-i--yogyakarta-2018.html |
| 12. | 40 | 40 | Puspitasari E. The dual role of women in working mothers in Pakembinangun, Pakem, Sleman, Yogyakarta [Peran ganda perempuan pada ibu bekerja di desa Pakembinangun, Pakem, Sleman, Yogyakarta]. Universitas Negeri Yogyakarta. 2016. Available: http://eprints.uny.ac.id/37957/ |
| 13. | 42 | 42 | Available: http://digilib.unisayogya.ac.id/338/ |
| 14. | 43 | 43 | doi:http://dx.doi.org/10.21927/ijnd.2019.7(3).89-96 |
| 15. | 45 |  | Remove |
| 16. | 53 | 52 | Available: http://eprints.ums.ac.id/86266/ |
| 17. | 56 | 55 | [Hubungan paparan asap dengan kejadian infeksi saluran pernapasan akut (ISPA) pada anak usia 0-5 tahun di wilayah pertanian Kecamatan Panti, Kabupaten Jember]  doi:10.14710/jekk.v5i2.7152 |
| 18. | 59 | 58 | [Evaluasi potensi mataair untuk kebutuhan air domestik di Kecamatan Cangkringan Kabupaten Sleman pasca erupsi Merapi 2010] |
| 19. | 60 | 59 | Available: http://www.slemankab.go.id/profil-kabupaten-sleman/geografi/karakteristik-wilayah |
| 20. | 64 | 63 | [Evaluasi kinerja PDAM Sleman di bidang operasi dan pelayanan pada ibukota Kecamatan Prambanan, Kalasan, Ngemplak, Bimomartani, Condong Catur]  Available: https://dspace.uii.ac.id/handle/123456789/16317 |
| 21. | 65 | 64 | [Hasil Survei Kualitas Air di Daerah Istimewa Yogyakarta 2015]  Available: https://www.bps.go.id/publication/2016/11/03/26dd424f1c7391a6c62adf33/hasil-survei-kualitas-air-di-daerah-istimewa-yogyakarta-tahun-2015.html |
| 22. | 68 | 67 | [Evaluasi pengendalian kinerja kualitas air minum pada depot air minum isi ulang di Kabupaten Sleman, Yogyakarta] |

**RESPONSES TO REVIEWERS**

Reviewer's Responses to Questions

**Comments to the Author**

Reviewer #1: Though, authors tried their level best to answer our queries, still there is room for improvement
a. there is lack of strong justification "why HDSS data is important for local policy makers"; does it mean that there is no other local data except HDSS to help the policy makers to make their decisions?

**Response:** Thank you. We have added the explanation that can be seen in line 88 – 90.

The Sleman HDSS is the only surveillance system in Sleman that collects data on population transitions, health status and social transitions periodically since 2015.

b. Very weak conclusion; 1st recommendation is related to immunization where authors mentioned that it is not related to ARI; 2nd recommendation is related to breastfeeding which is not supported by study findings/data

**Response:** Thank you for pointing this out. We have changed the conclusion. The changes can be seen in line 339 – 344.

Respondents who use tap water or refilled drinking water depots are at risk of experiencing ARI. The following Sleman HDSS survey must pay attention to the quality of water consumed by respondents, especially *E. coli* and nitrates. Further research needs to analyze the relationship between water quality and the incidence of ARI. It is to ensure the relationship between the use of water sources and the incidence of ARI in children under five so that the government can determine the right policy in reducing the morbidity of ARI in children under five.

We look forward to hearing from you in due time regarding our submission and to respond to any further questions and comments you may have.

Thanks for your kindly.

Best Regards,

Dr. Fathmawati Fathmawati
